# Adolescent Cyberbullying and Cyber Victimization: Longitudinal Study Before and During COVID-19

**DOI:** 10.2196/70508

**Published:** 2025-03-25

**Authors:** Peter Johannes Schulz, Marc-Olivier Boldi, Ann van Ackere

**Affiliations:** 1 Faculty of Communication, Culture and Society Università della Svizzera Italiana Lugano Switzerland; 2 Department of Communication & Media Ewha Womans University Seoul Republic of Korea; 3 Faculty of Business and Economics University of Lausanne Lausanne Switzerland

**Keywords:** cyberbullying and cyber victimization among adolescents, COVID-19, panel study, longitudinal data analysis, parental communication, exposure to violent media content

## Abstract

**Background:**

Adolescent cyberbullying has been a persistent issue, exacerbated by the shift to remote learning and increased screen time during the COVID-19 pandemic. These changes have sparked concerns about potential increases in cyberbullying and its associated risks.

**Objective:**

This study aims to explore how factors such as age, exposure to violent media, parental communication quality, internet access, sex, and sibling relationships influence cyberbullying behavior at school. Additionally, we examine how the COVID-19 pandemic may have altered these dynamics.

**Methods:**

Leveraging a panel dataset, we examine the same group of adolescents both before and during the pandemic. The analysis focused on identifying relationships between the selected factors and cyberbullying perpetration and victimization, with an emphasis on the dynamics introduced by the COVID-19 pandemic.

**Results:**

Perceived quality of parental communication was found to reduce the risk of both cyberbullying perpetration and victimization, with the former effect becoming more pronounced during the COVID-19 pandemic. Exposure to violent media increased both cyberbullying perpetration and victimization, but the effect on perpetration decreased during the COVID-19 pandemic. The well-established correlation between internet access and both cyberbullying perpetration and victimization remained unaffected by COVID-19. Surprisingly, adolescents with siblings were less likely to become victims or perpetrators of school-related cyberbullying, irrespective of the pandemic.

**Conclusions:**

In hindsight, COVID-19, functioning as a kind of natural experiment, has provided us with a unique opportunity to examine the effects of a global event, forcing major behavioral changes on the persistent challenge of cyberbullying in middle schools.

## Introduction

### Overview

The global issue of adolescents’ aggressive behavior has long been acknowledged as a significant concern, predating the outbreak of COVID-19. This pervasive issue has garnered considerable attention in the literature, with studies highlighting its profound impact on the well-being of young individuals [1-6]. With the advent of digital technologies and the widespread use of social media platforms, adolescents are increasingly exposed to online harassment, encompassing various forms of aggressive behaviors such as spreading rumors, sending threatening messages, or posting hurtful content. The consequences of cyberbullying extend beyond the digital realm, negatively affecting adolescents’ mental health, self-esteem, and overall psychosocial development. Research consistently links cyberbullying to heightened levels of anxiety, depression, and decreased self-esteem [7], as well as emotional distress among victims [3]. Moreover, the persistent nature of online harassment can lead to feelings of social isolation and a decline in academic performance [8-10]. The long-term consequences may manifest in a reluctance to engage in social interactions, thereby hindering the development of healthy peer relationships [11].

With the onset of COVID-19 and the subsequent surge in remote learning and screen time during the pandemic, concerns have emerged regarding the potential escalation of cyberbullying incidents. Within just 1 year after March 11, 2020, when the World Health Organization declared COVID-19 a pandemic, the first studies were published comparing the prevalence of cyberbullying perpetration and victimization before and during COVID-19. In 2023, the first systematic review [12] and meta-analysis on this comparison were published [13]. As summarized by Vaillancourt et al [2] in a recent overview, the results are contradictory, with studies reporting an increased, decreased, or stable trend in cyberbullying. Most of the studies involve comparing 2 cross-sectional surveys, one conducted before and one during the pandemic, with only a few comparisons based on panel data.

The objectives of this study are 3-fold. First, we aim to investigate whether and, if so, in which direction the trend of cyberbullying evolves with age. The second objective delves into several factors associated with changes in this trend. Specifically, we aim to explore the role of factors such as exposure to violent media content, the quality of parental communication, access to the internet, as well as the influence of sex and sibling relationships. The third objective is to assess the extent to which changes in the trend can be attributed to COVID-19: our unique time-series data enable us to do this by comparing the same adolescents in periods before and during the COVID-19 pandemic. The data collection for this study began in September 2017 and concluded in the summer of 2021, spanning 6 waves. While the richness and longitudinal nature of such a comprehensive panel study are clear advantages, they also present challenges: the data, particularly from the early waves, may appear outdated, and organizing such a large dataset requires substantial effort, making it challenging to publish findings immediately after data collection is completed.

### Literature Review and Formulation of Hypotheses

We review the literature on cyberbullying perpetration and victimization, with particular attention to those covering the COVID-19 period, to derive a set of hypotheses to achieve the objectives listed above.

#### Cyberbullying and COVID-19

To understand the impact of COVID-19 on the prevalence of aggressive behavior among adolescents, it is necessary to examine trends predating the pandemic. Cyberbullying perpetration is intrinsically related to cyberbullying victimization, which refers to the experience of being targeted and harmed by cyber aggression—those who engage in cyberbullying may themselves become victims of retaliatory behaviors [14,15].

Several studies on bullying patterns suggest that cyberbullying was already a concern before COVID-19 [16-18]. With the advent of digital technology and social media platforms, there has been a gradual increase in the incidence of cyberbullying. A study conducted in the United States spanning the period 2010-2017 reported a consistent growth in cyberbullying [19]. However, longitudinal studies tracking adolescents over periods longer than 6 to 12 months are scarce, limiting our understanding of the factors contributing to this upward trend [15,20]. Adolescents are believed to be particularly vulnerable to cyberbullying due to their frequent use of smartphones and social networking sites, which provide perpetrators with anonymity and easy access to their targets. Research conducted in the years preceding the pandemic underscored the detrimental effects on adolescents’ mental health, academic performance, and social relationships. A systematic review of cyberbullying studies conducted worldwide from the beginning of 2015 to December 2019 suggests a continuous increase—prevalence rates of cyberbullying victimization ranged from 6% to 46.3% in 2015, increasing to 13.99% to 57.55% in 2019 [21].

The role of age in adolescent cyberbullying presents mixed evidence. While certain studies have argued that both perpetration and victimization occur equally across different age groups [22], others have found that cyberbullying is particularly prevalent in middle schools, with incidents increasing after the fifth grade and peaking in the eighth grade [5,23,24]. One explanation for the mixed evidence might be that most studies are either cross-sectional or based on repeated survey designs.

There is an ongoing controversy regarding whether cyberbullying perpetration and victimization went up, remained stable, or decreased during the lockdown caused by COVID-19. While several studies have found an increase [18,25-29], a UNICEF report from 2020 indicates a 17% decrease in cyberbullying during the lockdown. Other studies report not only an overall decrease in cyberbullying perpetration, but also a decrease in victimization, at least for those adolescents with moderate social media use [30].

Besides the question of whether a real change occurred in both periods, and if so, in which direction, another query arises: if such a change did occur, is it due directly to the pandemic or to a shift in the main factors associated with cyberbullying before and during the pandemic? To address this issue, we include COVID-19 as an indicator variable to enable testing for interaction effects.

Considering the lack of longitudinal studies, it is not surprising that Vallaincourt et al [2] conclude that due to the predominantly cross-sectional design of existing studies, it is not possible to make definitive statements about true change. Given the inconclusive evidence regarding the impact of COVID-19 and the role of age, we formulate the following hypothesis:

Cyberbullying perpetration and victimization initially increase with age and then stabilize. The observed relationship is unchanged before and after COVID-19.

H1

#### Use of Information and Communication Technologies and Exposure to Violent Media Content

The use of information and communication technologies has long been recognized as a notable risk factor linked to cyberbullying perpetration and victimization [11,31]. Particularly for adolescents aged 12 to 17 years, social networking sites provide a platform where approximately 85% have reported witnessing negative interactions [32]. The amount of time adolescents spend using information and communication technologies represents another crucial factor [14,33], in particular the time of exposure to antisocial media content which, in turn, predicts that an adolescent is more likely to engage in cyberbullying over time [34]. A recent systematic review and meta-analysis comprising 46 studies found a remarkably strong increase in screen time of nearly 1.5 hours per day compared with pre–COVID-19 levels, constituting a surge of approximately 52%. Adolescents aged 12 to 18 years contributed significantly to this trend [35]. Applications like TikTok, Pinterest, Reddit, Facebook, Snapchat, Instagram, LinkedIn, and Twitter have seen an increase in active users, ranging from 8% to 38% [36]. Additionally, a study analyzing data from Twitter users in 16 countries and information gathered from Reddit in one country highlights a substantial growth in abusive content on both platforms [37]. Based on this, we formulate the next 2 hypotheses.

Increased access to the internet increases both the perpetration and victimization of cyberbullying. The observed relationship is unchanged before and after COVID-19.

H2

Increased exposure to violent media content increases both perpetration and victimization of cyberbullying. The observed relationship is unchanged before and after COVID-19.

H3

#### Parental Communication, Siblings, and Sex

Meta-analytic research has demonstrated that a positive family environment serves as a protective factor against bullying (and cyberbullying) perpetration and victimization [38-40]. Conversely, a perceived lack of quality in parental communication has been linked to instances of adolescent bullying and cyberbullying [38,41-43].

Some scholars posit that the shift to remote learning during COVID-19, combined with a stressful economic environment, has heightened the burden on families as they navigate the challenges of childcare, education, and professional challenges [13,44]. The resulting stress may negatively impact the quality of parental communication [45]. We thus formulate the following hypothesis:

Higher quality of parental communication decreases both the perpetration and victimization of cyberbullying. The observed relationship is unchanged before and after COVID-19.

H4

The impact of siblings on cyberbullying has not really been researched. Existing studies focus on bullying between siblings, rarely on the impact of siblings on bullying by schoolmates. For instance, Borualogo and Casas [46], in their study involving young adolescents aged 10-12 years in Indonesia, concluded that physical bullying between siblings increased during COVID-19 compared with prepandemic levels, while incidents of school bullying notably decreased. Given this lack of prior studies, we formulate the following explorative hypothesis:

Having siblings is a risk factor for cyberbullying perpetration and victimization at school. The observed relationship is unchanged before and after COVID-19.

H5

Concerning sex, certain studies [22,47] argue that this does not predict cyberbullying victimization, while others [1,30,48,49] conclude that boys report more perpetration and girls report more victimization. We formulate the following hypothesis:

There is a higher rate of cyberbullying perpetration and a lower rate of victimization among boys compared to girls; the difference persists with aging. The observed relationship is unchanged before and after COVID-19.

H6

## Methods

### Sample Participants and Procedure

Data for this research are drawn from a 6-wave panel survey conducted among middle school students in the Canton of Ticino, Switzerland, between 2017 and 2021. Ticino is the southernmost canton of Switzerland and the only one with Italian as the official language. It is considered one of the most prosperous regions in Switzerland and Europe [50]. On a global scale, Switzerland falls within the average range for bullying incidents in compulsory schools, as reported by the Programme for International Students Assessment (Organisation for Economic Co-operation and Development; 2019). Exactly 1 month after the World Health Organization had declared COVID-19 a pandemic on March 11, 2020, all middle schools of the Canton of Ticino were closed, and students transitioned to online learning modalities. Two months later, in May 2020, all students in compulsory school years returned to offline schooling.

At the start of the study in September 2017, a random sample consisting of 66% of all first-year public middle school classes across the 5 areas of the canton was selected. Private schools with more than 2 first-year classes were also invited to participate, and one of the 3 schools that met this criterion accepted the invitation. From this school, one of the 2 first-year classes was randomly selected for inclusion. Thus, the research encompassed 101 classes randomly chosen from 1 private and 35 public middle schools.

Data were collected through self-administered, paper-and-pencil questionnaires completed in class under the supervision of a teacher. The data from the 6 waves were matched using a unique identifier linked to each student’s name, which only collaborating school staff accessed for survey distribution purposes. To protect participants’ privacy, teachers were instructed to place all completed questionnaires in an envelope and seal it in front of the students. The remaining missing data were due to absences on the day of data collection or students moving out of the canton. Students who changed schools were retained in our sample as long as they moved to another school and class included in our sample. However, if they transferred to a school outside the selected sample, they were no longer included in the study.

For this research, data collected on 6 different occasions were analyzed: November and December 2017 (T1), May and June 2018 (T2), November and December 2018 (T3), May and June 2019 (T4), October and November 2020 (T5), and May and June 2021 (T6). The observation time thus ranged from the beginning of the ﬁrst grade to the end of the fourth grade. The average age was 13.22 (SD 1.33) years. At time point T1, ages ranged from 10.71 to 13.94 years, and at T6, from 14.21 to 17.44 years.

The first 4 waves took place before the COVID-19 pandemic, as initially scheduled. The last 2 had to be delayed due to the closure of schools in May 2020. The final random sample included 2052 students who were invited to participate in T1. Of these 2052 students, a total of 2022 (98.54%) participated at T1, 1879 (91.57%) at T2, 1896 (92.4%) at T3, 1865 (90.89%) at T4, 1871 (91.18%) at T5, and 1869 (91.08%) at T6.

### Ethical Considerations

The survey was conducted with the approval and collaboration of the Cantonal Department for School Affairs (Divisione della scuola e l’Ufficio dell’insegnamento) and involved a representative sample of students. In addition to authorization from the cantonal school authorities, parents were informed about the study and given the option to exclude their children (passive consent). Less than 4% of students (n=75) did not participate due to parental objections. The institutional research board of the University della Svizzera Italiana (USI) did not consider the study to require approval, provided that the school authorities in the Canton of Ticino and the parents of the adolescents had approved the study. Informed consent was obtained from all adolescents prior to their participation in the study (29-01-2019Ls). The consent process included a clear explanation of the study’s purpose, procedures, potential risks, and benefits, as well as assurances of confidentiality and the voluntary nature of participation. The adolescents were informed that they could stop filling in the questionnaire at any time. The study ensures the privacy and confidentiality of participants by maintaining all collected data in an anonymous or deidentified format. No personal identifiers are linked to the data. All study data are stored on servers with access restricted to authorized research personnel only, ensuring high level of privacy and confidentiality for participants. Participants did not receive any compensation for their participation in the study.

### Variable Selection

Table 1 provides an overview of all variables used in our analysis; it is followed by a description of each measure.

**Table 1:** Variable description.

Variable		Scale	Description
**Dependent variables**			
	Cybperp	1 (never) to 4 (often)	Perception of cyberbullying perpetration
	Cybvic	1 (low) to 4 (high)	Perception of cyberbullying victimization
**Independent variables**			
	Medexp	1 (never) to 5 (very often)	Exposure to violent media content
	Parqual	1 (disagree) to 5 (agree)	Perceived quality of communication with parents
	Siblings	0 or 1	Indicator variable: only child or 1 or more siblings
	Age	Years	Age of participants
	COVID	0 or 1	Indicator variable: before or after COVID-19
	Sex	0 or 1	Indicator variable: male or female
	Device	0 or 1	Indicator variable: no access (0) or access (1) to a device connected to internet
**Control variables**			
	Parents	0 or 1	Indicator variable: child lives with none or one (0) or both (1) parents
	Econf	1 (not at all well) to 5 (very well)	Self-reported (by students) economic status of household

#### Perpetration of Cyberbullying

Perpetration of cyberbullying (abbreviated as “Cybperp”) was assessed on all 6 occasions using 6 of the 11 items from the European Cyberbullying Intervention Project Questionnaire [51] to evaluate the perpetration of aggressive acts online. For more details regarding the multi-item measurement scale, refer to Bullo and Schulz [34]. Measurement invariance of cyber peer aggression was tested in a structural equation modeling framework using confirmatory factor analysis, which demonstrated that all 4 levels of measurement invariance (from configural factorial over weak and strong factorial to strict factorial invariance [52]) can be assumed for the data. Additional details are provided in Bullo and Schulz [34]. For each item, participants were asked to indicate how often—in the previous 6 months—they had engaged in these behaviors. The response scale went from “Never” (1) to “Often” (4). McDonald ω was used to assess the reliability of the measure, which ranged between 0.70 and 0.88.

#### Cyberbullying Victimization

Cyberbullying victimization (abbreviated as “Cybvic”) was measured using 6 items selected from the European Cyberbullying Intervention Project Questionnaire project. Participants reported the frequency with which they had been victims of various forms of cyber aggression, including direct verbal aggression (eg, being insulted via texts or web-based messages; being threatened), relational aggression (eg, being excluded in the virtual world; being a victim of badmouthing or gossiping), and media-related aggression (eg, unwanted sharing of embarrassing pictures or videos). The response scale ranged from “Never” (1) to “Often” (4). McDonald ω was used to assess the reliability of the measure, which ranged between 0.84 and 0.88.

#### Exposure to Violent Media Content

Exposure to violent media content (abbreviated as “Medexp”) was assessed by calculating an average score from 6 items regarding how often adolescents have watched media (including television, smartphones, video games, or the internet) depicting people fighting, destroying others’ property, stealing, shooting, taking drugs, or drinking alcohol (1=never to 5=very often). McDonald ω was used to assess the reliability of the measure, which ranged from 0.89 to 0.93.

#### Parental Communication Quality

Parental communication quality (abbreviated as “Parqual”) was measured using a slightly modified version of a scale developed by Guilamo-Ramos et al [53], which includes dimensions of perceived parental expertise, trustworthiness, and accessibility. The scale consists of 4 items rated on a 5-point scale from “Totally disagree” (1) to “Totally agree” (5), with higher values indicating higher perceived quality in adolescents talking to their parents. More details are provided in Bullo and Schulz [41]. Again, McDonald ω was applied to assess the reliability of the measure, which ranged from 0.60 to 0.75.

For all 4 multi-item measurement scales (cyberbullying perpetration and victimization, exposure to violent media content, and perceived parental communication quality), the unidimensionality of the measures was confirmed using confirmatory factor analysis [54].

#### Siblings

Furthermore, adolescents were asked how many brothers and sisters lived with them in their household. We used a 0 or 1 indicator, where 0 indicates an only child, and 1 indicates at least 1 sibling.

#### Age

This variable represents the adolescents’ age, expressed in years.

#### COVID

This 0 or 1 indicator variable captures for each wave whether it took place before (0) or after (1) the school closure due to the COVID-19 pandemic.

#### Sex

This is an indicator variable, where 0 indicates male and 1 female.

#### Access to Communication Devices

Access to communication devices (abbreviated as “Device”) was assessed by asking participants whether they possess a cellphone with or without internet access, a tablet or computer with internet access, a PlayStation, and whether they have a television in their room. The measure of access was defined as a 0 or 1 indicator, a value of 1 indicating that the child has access to at least 1 device with internet access.

#### Parental Status

Parental status (abbreviated as “Parents”) was assessed by asking adolescents whether they lived with both, one, or none of their parents. We used a 0 or 1 indicator, where 0 indicates that they live with at most one of their parents, and 1 indicates they live with both parents.

#### Perceived Economic Status of the Family

The perceived economic status of the family (abbreviated as “Econf”) was assessed during the first data collection by asking students to indicate how financially well off their family is on a scale from “Not well at all” (1) to “Very well” (5).

#### Descriptive Statistics

Table 2 presents a demographic profile of the sample, together with means, SDs, and indices of skewness and kurtosis for key variables used in the formal models we tested.

**Table 2:** Descriptive statistics. Longitudinal assessment of cyberbullying perpetration, cyberbullying victimization, violent media exposure, perceived quality of parental communication, siblings, age, sex, parental status, internet access, and self-reported economic status of the household.

Statistics			T1		T2	T3		T4		T5		T6
**Cyberbullying perpetration (Cybperp)**												
	Participants, n	1471		1440		1425	1409		1067		988	
	Mean (SD)	1.10 (0.21)		1.20 (0.36)		1.17 (0.34)	1.25 (0.43)		1.26 (0.39)		1.24 (0.44)	
	Skewness (SE)	0.21		0.36		0.34	0.43		0.39		0.44	
	Kurtosis (SE)	3.86		2.84		4.09	2.44		2.60		2.70	
**Cyberbullying victimization (Cybvic)**												
	Participants, n	1454		1433		1419	1402		1060		989	
	Mean (SD)	1.15 (0.31)		1.29 (0.45)		1.19 (0.38)	1.34 (0.53)		1.23 (0.39)		1.28 (0.46)	
	Skewness (SE)	0.31		0.45		0.38	0.53		0.39		0.46	
	Kurtosis (SE)	3.56		2.04		3.17	2.03		2.79		2.31	
**Violent media exposure (Medexp)**												
	Participants, n	1475		1440		1430	1413		1068		994	
	Mean (SD)	1.58 (0.82)		2.05 (1.05)		1.95 (1.06)	2.43 (1.16)		2.57 (1.16)		2.74 (1.17)	
	Skewness (SE)	0.82		1.05		1.06	1.16		1.16		1.17	
	Kurtosis (SE)	1.76		0.90		1.08	0.51		0.34		0.12	
**Perceived quality of parental communication (Parqual)**												
	Participants, n	1441		1445		1434	1414		1059		989	
	Mean (SD)	2.99 (0.89)		3.03 (0.82)		3.09 (0.81)	3.01 (0.85)		2.85 (0.83)		2.84 (0.83)	
	Skewness (SE)	0.89		0.82		0.81	0.85		0.83		0.83	
	Kurtosis (SE)	–0.50		–0.59		–0.71	–0.74		–0.42		–0.41	
**Siblings**												
	Participants, n	1480		1457		1448	1423		1070		997	
	Mean	0.82		0.81		0.80	0.79		0.79		0.79	
**Age (years)**												
	Participants, n	1497		1499		1498	1497		1506		1434	
	Mean (SD)	11.53 (0.40)		12.03 (0.40)		12.53 (0.40)	13.03 (0.40)		14.53 (0.40)		15.03 (0.40)	
**Sex**												
	Participants, n	1496		1499		1498	1496		1506		1001	
	Mean	0.49		0.49		0.49	0.49		0.49		0.50	
**Parental status (Parents)**												
	Participants, n	1480		1457		1449	1423		1070		996	
	Mean	0.79		0.77		0.77	0.75		0.74		0.74	
**Internet access (Device)**												
	Participants, n	1339		1215		1197	1231		888		933	
	Mean	0.80		0.84		0.90	0.93		0.98		0.99	
**Self-reported economic status of household (Econf)**												
	Participants, n	1435		1435		1435	1435		1435		1370	
	Mean (SD)	3.86 (0.70)		3.86 (0.70)		3.86 (0.70)	3.86 (0.70)		3.86 (0.70)		3.86 (0.69)	

### Statistical Methods

Two mixed-effect models were set up for the 2 dependent variables, namely “Cybperp,” and “Cybvic.” The independent variables were “Age,” “COVID,” “Sex,” “Siblings,” “Medexp,” “Parqual,” and “Device.” For the variable age, a polynomial of order 3 was fitted to test the variation of the effect. The 2-way interactions between “COVID” and the other variables were included, as well as between “Age” and “Sex.” The 3-way interaction between “COVID,” “Age,” and “Sex” was also included. A random intercept per participant was included in the model to account for the repeated measure framework. Finally, “Econf” and “Parents” were included as control variables. The models were fitted using the statistical software R (R Foundation for Statistical Computing) and its package *lme4* [55]. Multiple comparisons were performed using package *emmeans* [56] and Sidak-adjusted *P* values. Once fitted, a selection of variables was performed by removing the nonsignificant ones according to ANOVA *F* tests. These results are given in Tables S1 and S2 in Multimedia Appendix 1. Missing data cases were discarded. Cases were fixed based on the most complete model before the model selection. For the analysis, complete cases for the selected model’s features were used.

## Results

### Overview

The victimization and perpetration model coefficients are given in Tables S3 and S4 in Multimedia Appendix 1. To test H1 and H6, which involve age, we set the comparisons at the mean age, minus and plus 1 SD, that is, at 11.8, 13.1, and 14.5 years. The results are presented below, per hypothesis, first for victimization and then for perpetration.

### Hypothesis 1

#### Victimization

The hypothesis is validated for female individuals, but only partially for male individuals (Table S5 in Multimedia Appendix 1).

Indeed, for female individuals, the observed increase in victimization by “Age” is significant between 11.8 and 13.1 years (≈0.07, *z*=3.83, *P<*.001) and nonsignificant between 13.1 and 14.5 years (≈0.03, *z*=0.88, *P=*.62). For male individuals, this increase is significant in both cases (≈0.13, *z*=7.17, *P<*.001; ≈0.16, *z*=4.98, *P<*.001). As expected, there is no difference between before and after COVID-19, as no interaction involving “COVID” and “Age” is significant.

#### Perpetration

The hypothesis is validated for female and male individuals (Table S6 in Multimedia Appendix 1). Indeed, the observed increase in perpetration by “Age” is significant between 11.8 and 13.1 years (male: ≈0.04, *z*=3.53, *P<*.001; female: ≈0.04, *z*=3.04, *P<*.01) and nonsignificant between 13.1 and 14.5 years (male: ≈0.03, *z*=1.59, *P=*.21; female: ≈0.00, *z*=–0.08, *P*>.99). As expected, there is no difference between before and after COVID-19, as no interaction involving “COVID” and “Age” is significant.

### Hypothesis 2

#### Victimization

The hypothesis is validated as the difference in victimization between with and without access to the internet is significant (≈0.09, *z*=5.03, *P<*.001; Table S7 in Multimedia Appendix 1). Without significant interaction between “COVID” and “Device,” this effect remains unchanged, as expected.

#### Perpetration

The hypothesis is validated as the difference in perpetration between with and without access to the internet is significant (≈0.07, *z*=4.48, *P<*.001; Table S8 in Multimedia Appendix 1). Without significant interaction between “COVID” and “Device,” this effect remains unchanged as expected.

### Hypothesis 3

#### Victimization

The hypothesis is validated. Indeed, the effect of “Medexp” is significant and positive (≈0.06, *t*_5791_=11.7, *P<*.001; Table S9 in Multimedia Appendix 1). Without significant interaction between “Medexp” and “COVID,” this effect remains unchanged as expected.

#### Perpetration

The hypothesis is validated. The effect of “Medexp” is significant before and after COVID-19 (before: ≈0.09, *z*=16.88, *P<*.001; after: ≈0.07, *z*=10.32, *P<*.001; Table S10 in Multimedia Appendix 1). Although the effect remains significant and positive before and after COVID-19, the difference in the slope is mildly significant (≈0.02, *z*=2.06, *P=*.04; Table S11 in Multimedia Appendix 1). Thus, the effect of “Medexp” on cyberbullying perpetration decreased after COVID-19, contrary to our expectations.

### Hypothesis 4

#### Victimization

The hypothesis is validated. The effect of “Parqual” is significant and negative (≈–0.06, *t*_5789_=–8.9, *P<*.001; Table S9 in Multimedia Appendix 1). Without interaction between “COVID” and “Parqual,” the relationship did not change after COVID-19.

#### Perpetration

The hypothesis is validated. Both before and after COVID-19, the effect of “Parqual” is negative and significant (before: ≈–0.02, *z*=–4.00, *P<*.001; after: ≈–0.05, *z*=–5.20, *P<*.001; Table S12 in Multimedia Appendix 1). In addition, contrary to our expectation, this deterring effect significantly increased after COVID-19 (≈0.03, *z*=2.34, *P=*.02; Table S13 in Multimedia Appendix 1).

### Hypothesis 5

#### Victimization

The hypothesis is not validated. The effect of “Siblings” is significant and negative (≈–0.05, *t*_2640_=–2.80, *P<*.01; Table S9 in Multimedia Appendix 1). Contrary to our expectations, the data indicate that having siblings is a protective factor: those with siblings are less likely to become victims of cyberbullying at school compared with those without siblings. As there is no significant interaction between “Siblings” and “COVID,” this effect is the same before and after COVID-19.

#### Perpetration

The hypothesis is not validated. As for victimization, the effect of “Siblings” is significant and negative (≈–0.03, *t*_2714_=–2.26, *P=*.02; Table S14 in Multimedia Appendix 1). Again, contrary to our expectations, the data indicate that having siblings is a deterring factor. Without significant interaction between “Siblings” and “COVID,” that effect is the same before and after COVID-19.

### Hypothesis 6

#### Victimization

The hypothesis is partially validated. The expected lower rate for male individuals is mildly significant at early age before COVID-19 (male – female; age 11.8 years; ≈–0.05, *z*=–2.78, *P=*.03; Table S15 in Multimedia Appendix 1) but not afterward (male – female; age 11.8 years; ≈–0.19, *z*=–2.63, *P=*.05). At any other age, before and after COVID-19, differences were not significant.

#### Perpetration

The hypothesis is not validated. There is no difference between male and female individuals at any age (Table S16 in Multimedia Appendix 1). In addition, without interaction between “COVID” and “Age” and between “COVID” and “Sex,” this nonrelation remains the same before and after COVID-19.

## Discussion

### Principal Findings

The focus of our study is on the question of whether COVID-19 has changed the relationship between cyberbullying and several of its drivers, among adolescents. We were particularly interested in whether COVID-19 has made these relationships more pronounced, whether they have remained unchanged, or whether they have been mitigated. These questions are not primarily important because of the current demand for an analysis of COVID-19 and its impact on social life [57]. Rather, it is also expected that an unforeseen event like COVID-19 could lead to a better understanding of the factors associated with the increasing challenge of cyberbullying in schools [58]. Ultimately, this also involves the expectation of gaining a better understanding of the phenomenon of cyberbullying among adolescents.

Regarding our first hypothesis, namely the increase in cyberbullying victimization among adolescents with age, our data confirm this effect, known from previous studies, at least for the early middle school years, when adolescents are between 11 and 13 years old. This effect applies equally to girls and boys. However, from the age of 13 years onward, this seems to change, as the increase in cyberbullying victimization among girls is no longer significant, while cyberbullying perpetration among boys continues to grow.

When interpreting these data, it is important to note that the end of the increasing trend in cyberbullying victimization coincides with the onset of COVID-19. Therefore, it is difficult to determine to what extent the end of the increase among girls or the continuing upward trend among boys is attributable to age and how much is due to the onset of COVID-19. Previous studies on cyberbullying victimization have shown that this trend ends at a certain age among adolescents. It is also challenging to comment on the magnitude of this effect. In other words, it is unclear how strong the increase in cyberbullying victimization among boys would have been if COVID-19 had not occurred.

As to our second hypothesis, there is a well-documented correlation, supported by numerous studies [59], between adolescents owning devices that provide internet access and cyberbullying perpetration and victimization: when adolescents have access to a device that allows them to go online, the likelihood of them becoming a victim or perpetrator of cyberbullying rises. This correlation was observed in our study as well, both before and during the outbreak of COVID-19, with no significant change in the strength of this relationship during the pandemic.

It is important to consider that even without personal internet-enabled devices, adolescents can still fall victim to or engage in cyber aggression, for example, by using internet access on school computers. Moreover, by the end of middle school, only a small number of students in our study still lacked a device that enabled them to access the internet. Nevertheless, the lack of a personal internet-enabled device remains a protective factor, regardless of the age at which adolescents acquire such a device.

Regarding the relationship between exposure to violent media content and the perpetration or victimization of cyberbullying among adolescents, previous meta-analytical studies [14,20,24,60,61] provide strong evidence supporting this link. Children highly exposed to violent media content (“Medexp”=5) have on average a victimization score of 0.25 larger than nonexposed children (“Medexp”=1). This is a major increase, as the average “Cybvic” score ranges between 1.15 and 1.34 for the different data-collection waves. The impact of violent media exposure is even larger for the perpetrators: a score increase of 0.33 and 0.28 before and during COVID-19, respectively, when “Medexp” increases from 1 to 5, compared with average scores ranging between 1.10 and 1.26.

The debate often centers on how violent media content leads to such behaviors, whether through imitation or other mechanisms [20,61]. Therefore, it is not surprising that our study also confirms this association. Longitudinal studies do not provide insights into causal relationships between exposure to violent media content and cyberbullying perpetration or victimization. However, it is notable that the effect of violent media exposure on cyberbullying remains substantial and persistent over the 4 years of our study. Additionally, this effect appears to diminish during COVID-19. This reduction cannot be explained by the age-related decrease in cyberbullying tendencies discussed in hypothesis 1, but rather seems to be an interaction effect that lessens during the pandemic. In this sense, one might consider it a positive effect of COVID-19: the pandemic has reduced the impact of exposure to violent media content on aggressive behavior. The exact reasons for this reduction in the relationship between exposure to violent media and cyberbullying victimization or perpetration, before and during COVID-19, remain unclear, but could be understood through Social Learning Theory: during COVID-19, changes in social contexts (eg, increased time spent at home and less face-to-face peer interaction) might have altered how adolescents process and enact behaviors learned from violent media. The reduced opportunities for social interaction and confrontation in physical spaces may have diminished the translation of media-inspired aggression into actual cyberbullying acts.

Hypotheses 2 and 3 are not unrelated, as access to internet and exposure to violent media are intrinsically linked. The finding that possessing an internet-enabled device consistently increases the likelihood of adolescents becoming victims or perpetrators of cyberbullying, with no significant change before and during COVID-19, can be explained by the general aggression model [24,62-64], which posits that exposure to aggressive stimuli (including violent media or environments) can increase aggressive thoughts, feelings, and behaviors. Access to the internet via a device provides a platform where aggressive behavior can be exhibited. The presence of a device enables easier access to both aggressive content and opportunities for cyberbullying, maintaining a consistent risk factor for both victimization and perpetration regardless of external circumstances like the pandemic.

Another possible explanation comes from Cohen and Felson’s [65] routine activity theory [66], which suggests that the changes in routine activities, such as increased parental supervision, reduced peer interaction, and changes in web-based activities, may have disrupted the conditions that facilitate cyberbullying perpetration. The decrease in the effect of violent media on perpetration could be because adolescents were less motivated to act aggressively or had fewer opportunities to engage in cyberbullying due to increased monitoring and altered social dynamics.

Our hypothesis 4, namely the assumption that a higher quality relationship with parents serves as a protective factor for adolescents, making them less likely to fall victim to or become a perpetrator of cyber aggression, has largely been confirmed. Interestingly, the protective effect of positive parental communication in preventing adolescents from becoming victims of cyber aggression is comparable in magnitude to the harmful impact of exposure to violent media content. On average, an increase of 1 unit in exposure to violent media content can be offset by an equivalent increase in the perceived positive quality of the relationship between adolescents and their parents.

What is particularly noteworthy, however, is that a positive relationship with parents not only acts as a protective factor against becoming a victim of cyber aggression but also seems to have a (more limited) deterrent effect on adolescents’ aggressive behavior toward others in cyberspace. This deterrent effect, although inferior to the impact of exposure to violent media, doubled during the COVID-19 crisis compared with the period before the pandemic.

We suggest that this finding can be explained through various theoretical perspectives. First, social control theory [67] posits that strong social bonds, such as those with family, serve as a deterrent to deviant behavior, including cyberbullying. During the pandemic, adolescents may have experienced increased stress, uncertainty, and isolation. In such a context, the role of positive communication with parents likely became more crucial in maintaining social control and discouraging deviant behavior. The intensification of family interactions due to lockdowns and restrictions may have strengthened these social bonds, leading adolescents to rely more on their parents for emotional support and guidance, thereby enhancing the protective effect against cyberbullying.

Another explanation comes from stress and coping theory, which posits that individuals cope with stress in various ways, including seeking support from social relationships [68]. Adolescents who could communicate positively with their parents may have experienced better emotional regulation and lower stress levels, reducing the likelihood of resorting to cyberbullying as a maladaptive coping strategy [69].

Regarding our fifth hypothesis, the results were contrary to our expectations: the data indicate that having siblings acts as a protective factor. Adolescents with siblings are less likely to become victims of cyberbullying at school than those without siblings. The magnitude of this protective effect is slightly lower than that observed in our previous hypotheses, suggesting that having siblings is, on average, equivalent to improving parental communication or decreasing exposure to violent media content by a bit less than 1 point on their respective scales. Similarly, our data show that having siblings also acts as a deterrent against becoming a cyberbully, although to a lesser extent, comparable to an increase of 1 point in the quality of parental communication, but much less than a decrease of 1 point in media exposure. We found no change in the effect of having siblings before and after the COVID-19 pandemic.

One possible explanation for these findings comes from the buffering hypothesis in social support theory [70,71], which posits that social relationships can buffer the impact of stressful events. Within this framework, siblings can offer emotional support, companionship, and a sense of belonging, which may protect against the isolation that can make a child more vulnerable to bullying [72].

Finally, based on findings from similar studies [73-75], we hypothesized that boys would exhibit a higher rate of cyberbullying perpetration, and a lower rate of victimization, compared with girls, with these differences persisting as they age. However, the lower rate of victimization among boys was only observed in the very early stage when they were around 11 years old. As they aged, the difference in victimization rates between boys and girls diminished and had already disappeared before the onset of COVID-19. Regarding the perpetration rate, no significant difference was found between male and female individuals.

### Concluding Remarks

In summary, we can conclude that COVID-19 likely had a limited overall impact on both cyberbullying perpetration and victimization. Moreover, while certain factors were identified as either increasing or decreasing the likelihood of cyberbullying, COVID-19 only partially influenced these relationships.

However, in certain cases, this impact is noteworthy. On the one hand, the perceived positive relationship with parents reduces both the risk of becoming a victim of cyberbullying and the likelihood of exhibiting aggressive behavior toward other adolescents, with the latter effect becoming more pronounced during COVID-19 compared with the period before. Notably, a positive relationship with parents served as a protective factor, reducing both the risk of cyberbullying victimization and the likelihood of engaging in cyberbullying. This protective effect was even stronger during the pandemic. These findings reaffirm previous research highlighting the role of family in mitigating cyberbullying risks [44] and emphasize its importance during times of crisis, such as COVID-19.

On the other hand, exposure to violent media was associated with increased rates of both cyberbullying perpetration and victimization, but interestingly, the effect of cyberbullying perpetration lessened during the pandemic. This suggests that while increased violent media consumption during an unexpected crisis may contribute to cyberbullying victimization, it does not necessarily translate into a rise in web-based aggressive behaviors. Additionally, an unexpected finding of our study revealed that adolescents with siblings were less likely to be involved as either victims or perpetrators of cyberbullying, independent of the pandemic’s influence.

The question of whether COVID-19 impacted cyberbullying behavior has been widely discussed in numerous studies. A recent systematic review and meta-analysis [13] concluded that, based on pooled prevalence rates, the pandemic led to a significant reduction in overall cyberbullying perpetration. However, as the authors caution, the lack of longitudinal studies limits the ability to draw definitive conclusions about the underlying factors driving these changes. Only a few studies, such as those by Huang et al [13] and Vaillancourt et al [2], have compared cyberbullying behaviors in the same group of participants before and during COVID-19, and even then, these studies involved only 2 or 3 waves of data collection.

In contrast, our study, which spans 6 waves—4 before and 2 during the pandemic—offers a more comprehensive picture. By tracking the same participants over time, we were able to estimate both the baseline rates of cyberbullying perpetration and victimization and the changes in these behaviors before and during the pandemic. Furthermore, we examined key contributing factors that either increase the likelihood of cyberbullying or act as protective measures. A particular strength of our panel study is the consistent use of the same measures for both cyberbullying outcomes and predictors across all 6 waves, allowing for a more robust analysis of trends over time.

### Limitations

Our study has several limitations due to the specific nature of our dataset. First, the sudden outbreak of the COVID-19 pandemic disrupted our planned data collection. While we were able to gather data from students at the start and end of each school year during the 4 pre–COVID-19 phases, this regularity was not maintained once the pandemic began. Data collection only resumed when students returned to school, resulting in an unbalanced distribution of waves between the prepandemic and postpandemic periods.

Second, our analysis is at a cohort level. We did not examine whether cyberbullying victims and perpetrators belong to specific groups, nor did we analyze the extent to which victims and perpetrators might be the same individuals, possibly at different points in time.

Finally, a strength of our dataset is that it is a panel study, following the same people over several years. The study includes 4 pre–COVID-19 waves (participants aged roughly 11-15 years) and 2 post–COVID-19 waves (participants aged roughly 14-17 years). The drawback is that we have few older adolescents before COVID-19 and few younger ones after COVID-19, which may be the cause of the lack of significance (large CIs) of certain age-related hypotheses.

In hindsight, COVID-19, functioning as a kind of natural experiment, has provided us with a unique opportunity to examine the effects of a global event, forcing major behavioral changes, on the persistent challenge of cyberbullying in middle schools.

## Appendix

Multimedia Appendix 1 Supplementary tables.

## Data Availability

The data supporting the findings of this study are available from the corresponding author upon reasonable request.
