# Supplementary material for: Adolescent Cyberbullying and Cyber Victimization: Longitudinal Study Before and During COVID-19
Source: J Med Internet Res. 2025 Mar 25;27:e70508. doi: 10.2196/70508 (PMC11979530; doi:10.2196/70508)
Supplement: Multimedia Appendix 1 [file jmir_v27i1e70508_app1.docx]

**Appendix**

**Table S1.** F-test based variable selection model of victimization

| Victimization |  |  |  |  |
| --- | --- | --- | --- | --- |
| Factor | F | df1 | df2 | P |
| Parents | 0.219 | 1 | 2614.8 | .640 |
| Econf | 0.122 | 1 | 1367.3 | .727 |
| Covid:poly(Age,3):Sex | 2.398 | 3 | 5682.9 | .066 |
| Covid:Device | 0.005 | 1 | 5957.7 | .941 |
| Covid:Parqual | 0.113 | 1 | 5847.9 | .737 |
| Covid:poly(Age,3) | 1.148 | 3 | 5915.7 | .328 |
| Covid:Siblings | 1.849 | 1 | 5593.2 | .174 |
| Covid: Medexp | 2.669 | 1 | 5809.1 | .102 |

**Table S2.** F-test based variable selection model of perpetration

| Perpetration |  |  |  |  |  |
| --- | --- | --- | --- | --- | --- |
| Factor |  | F | df1 | df2 | P |
| Parents |  | 0.545 | 1 | 2671.5 | .460 |
| Econf |  | 0.240 | 1 | 1363.9 | .624 |
| Covid:poly(Age,3):Sex |  | 0.811 | 3 | 5913.3 | .488 |
| Covid:Device |  | 0.006 | 1 | 5934.4 | .935 |
| Covid:poly(Age,3) |  | 0.745 | 3 | 5929.3 | .525 |
| Covid:Sex |  | 1.811 | 1 | 6301.6 | .178 |
| Covid:Siblings |  | 2.891 | 1 | 5601.3 | .089 |

**Table S3**. Mixed effect model for Cyber victimization

| **Victimization** | | | | | |
| --- | --- | --- | --- | --- | --- |
| Predictors | Est. | std. Error | df | t value | P |
| (Intercept) | 1.30 | 0.04 | 5,199.42 | 35.62 | **<.001** |
| Covid [Covid_1] | -0.28 | 0.05 | 6,288.23 | -6.12 | **<.001** |
| Age [1st degree] | 10.48 | 1.69 | 6,305.35 | 6.22 | **<.001** |
| Age [2nd degree] | 0.43 | 0.73 | 5,785.91 | 0.59 | .555 |
| Age [3rd degree] | -1.56 | 0.67 | 5,347.15 | -2.31 | **.021** |
| Sex [F] | -0.02 | 0.02 | 4,186.38 | -1.09 | .278 |
| Siblings | -0.05 | 0.02 | 2,640.40 | -2.80 | **.005** |
| Medexp | 0.06 | 0.01 | 5,791.20 | 11.74 | **<.001** |
| Parqual | -0.06 | 0.01 | 5,788.68 | -8.89 | **<.001** |
| Device | 0.09 | 0.02 | 6,088.18 | 5.03 | **<.001** |
| Age [1st degree] × Sex[F] | -7.10 | 2.35 | 6,316.2 | -3.03 | **.002** |
| Age [2nd degree] × Sex[F] | -1.94 | 1.02 | 5,797.75 | -1.91 | .057 |
| Age [3rd degree] × Sex[F] | 1.49 | 0.94 | 5,300.55 | 1.59 | .112 |
| covid [Covid_1] × Sex [F] | 0.14 | 0.06 | 6,277.83 | 2.17 | **.030** |
| **Random Effects** | | | | | |
| σ^2^ | 0.13 | | | | |
| τ_00_ _id_ | 0.04 | | | | |
| ICC | 0.25 | | | | |
| N _id_ | 1,403 | | | | |
| Observations | 6,331 | | | | |
| Marginal R^2^ / Conditional R^2^ | 0.071 / 0.303 | | | | |

**Table S4.** Mixed effect model for Cyber perpetration

| **Perpetration** | | | | | |
| --- | --- | --- | --- | --- | --- |
| Predictors | Est. | std. Error | df | t value | P |
| (Intercept) | 1.06 | 0.03 | 5,418.13 | 34.11 | **<.001** |
| Covid [Covid_1] | 0.07 | 0.05 | 6,129.33 | 1.41 | .158 |
| Age [1st degree] | 3.50 | 1.03 | 6,348.6 | 3.39 | **.001** |
| Age [2nd degree] | -0.33 | 0.53 | 5,677.35 | -0.62 | .533 |
| Age [3rd degree] | 0.69 | 0.52 | 5,575.97 | 1.33 | .185 |
| Sex [F] | -0.01 | 0.01 | 1,458.69 | -0.69 | .490 |
| Siblings | -0.03 | 0.01 | 2,714.40 | -2.26 | **.024** |
| Medexp | 0.09 | 0.01 | 6,162.51 | 16.88 | **<.001** |
| Parqual | -0.02 | 0.01 | 6,154.63 | -4.00 | **<.001** |
| Device | 0.07 | 0.02 | 6,151.26 | 4.48 | **<.001** |
| Age [1st degree] × Sex[F] | -2.61 | 0.68 | 5,857.03 | -3.84 | **<.001** |
| Age [2nd degree] × Sex[F] | -1.20 | 0.64 | 5,435.37 | -1.86 | .063 |
| Age [3rd degree] × Sex[F] | -0.95 | 0.68 | 5,807.27 | -1.40 | .161 |
| covid [Covid_1] × Medexp | -0.02 | 0.01 | 5,816.55 | -2.06 | **.039** |
| covid [Covid_1] × Parqual | -0.03 | 0.01 | 5,855.62 | -2.34 | **.019** |
| **Random Effects** | | | | | |
| σ^2^ | 0.08 | | | | |
| τ_00_ _id_ | 0.03 | | | | |
| ICC | 0.26 | | | | |
| N _id_ | 1404 | | | | |
| Observations | 6,364 | | | | |
| Marginal R^2^ / Conditional R^2^ | 0.102 / 0.335 | | | | |

**Table S5.** Tests related to Hypothesis 1 – Victimization

| H1 Cybvic | estimate | SE | z.ratio | adjusted P |
| --- | --- | --- | --- | --- |
| Sex = M |  |  |  |  |
| Age13.1 – Age11.8 | 0.1251 | 0.017 | 7.168 | <.001 |
| Age14.5 – Age13.1 | 0.1605 | 0.032 | 4.981 | <.001 |
| Sex = F |  |  |  |  |
| Age13.1 – Age11.8 | 0.0661 | 0.017 | 3.830 | <.001 |
| Age14.5 – Age13.1 | 0.0278 | 0.032 | 0.877 | .616 |

P-value adjustment by Sidak for the 2 tests

**Table S6**. Tests related to Hypothesis 1 – Perpetration

| H1 Cybperp | estimate | SE | z.ratio | adjusted P |
| --- | --- | --- | --- | --- |
| Sex = M |  |  |  |  |
| Age13.1 - Age11.8 | 0.0437 | 0.012 | 3.531 | <.001 |
| Age14.5 - Age13.1 | 0.0316 | 0.020 | 1.585 | .213 |
| Sex = F |  |  |  |  |
| Age13.1 - Age11.8 | 0.0377 | 0.012 | 3.041 | .005 |
| Age14.5 - Age13.1 | -0.0016 | 0.020 | -0.080 | .996 |

P-value adjustment by Sidak for the 2 tests

**Table S7.** Tests related to Hypothesis 2 – Victimization

| H2 Cybvic | estimate | SE | z.ratio | P |
| --- | --- | --- | --- | --- |
| Device0 – Device1 | -0.093 | 0.019 | -5.034 | <.001 |

**Table S8**. Tests related to Hypothesis 2 – Perpetration

| H2 Cybperp | estimate | SE | z.ratio | P |
| --- | --- | --- | --- | --- |
| Device0 - Device1 | -0.0681 | 0.015 | -4.481 | <.001 |

**Table S9.** Tests related to Hypotheses 3, 4 and 5 – Victimization

| H3,4,5, Cybvic | Estimate | SE | df | t-value | P |
| --- | --- | --- | --- | --- | --- |
| Medexp | 0.0626 | 0.005 | 5790 | 11.744 | <.001 |
| Parqual | -0.0593 | 0.007 | 5790 | -8.894 | <.001 |
| Siblings | -0.4560 | 0.016 | 2640 | -2.803 | .005 |

**Table S10.** Tests on slopes related to Hypothesis 3 – Perpetration

| *H3 Cybperp* | Medexp.trend | SE | z.ratio | adjusted *P* |
| --- | --- | --- | --- | --- |
| Covid_0 | 0.0872 | 0.005 | 16.883 | <.001 |
| Covid_1 | 0.0707 | 0.007 | 10.318 | <.001 |

p-value adjustment by Sidak for the 2 tests

**Table S11.** Test on slope difference related to Hypothesis 3 – Perpetration

| *H3 Cybperp* | estimate | SE | z.ratio | *P* |
| --- | --- | --- | --- | --- |
| Covid_0 - Covid_1 | 0.0165 | 0.008 | 2.061 | 0.039 |

**Table S12.** Tests on slopes related to Hypothesis 4 – Perpetration

| *H4 Cybperp* | Parqual.trend | SE | z.ratio | adjusted *P* |
| --- | --- | --- | --- | --- |
| Covid_0 | -0.0247 | 0.006 | -3.996 | <.001 |
| Covid_1 | -0.0498 | 0.010 | -5.202 | <.001 |

*P*-value adjustment by Sidak for the 2 tests

**Table S13**. Test on slope difference related to Hypothesis 4 – Perpetration

| *H4 Cybperp* | estimate | SE | z.ratio | *P* value |
| --- | --- | --- | --- | --- |
| Covid_0 - Covid_1 | 0.0251 | 0.011 | 2.340 | .019 |

**Table S14**. Tests related to Hypothesis 5 – Perpetration

| *H5 Cybperp* | Estimate | SE | df | t-value | *P* |
| --- | --- | --- | --- | --- | --- |
| Siblings | -0.0304 | 0.014 | 2710 | -2.257 | .024 |

**Table S15.** Tests related to Hypothesis 6 – Victimization

| *H5 Cybvic* |  |  |  |  |  |  |
| --- | --- | --- | --- | --- | --- | --- |
| Age | Covid | contrast | estimate | SE | z.ratio | adjusted *P* |
| 11.8 | Covid_0 | M - F | -0.0509 | 0.018 | -2.777 | .033 |
| 13.1 | Covid_0 | M - F | 0.0080 | 0.021 | 0.377 | .999 |
| 14.5 | Covid_0 | M - F | 0.1408 | 0.057 | 2.456 | .081 |
| 11.8 | Covid_1 | M - F | -0.1912 | 0.073 | -2.631 | .050 |
| 13.1 | Covid_1 | M - F | -0.1322 | 0.056 | -2.353 | .107 |
| 14.5 | Covid_1 | M - F | 0.0005 | 0.024 | 0.020 | >.999 |

*P*-value adjustment by Sidak for the 6 tests

**Table S16**. Tests related to Hypothesis 6 – Perpetration

| *H6 Cybperp* |  |  |  |  |  |
| --- | --- | --- | --- | --- | --- |
| Age | contrast | estimate | SE | z.ratio | adjusted *P* |
| 11.8 | M - F | -0.0099 | 0.015 | -0.680 | .872 |
| 13.1 | M - F | -0.0039 | 0.015 | -0.258 | .992 |
| 14.5 | M - F | 0.0293 | 0.018 | 1.638 | .275 |

*P*-value adjustment by Sidak for the 3 tests
